# F242. CHILDHOOD ADVERSITY AND PSYCHOTIC EXPERIENCES IN THE GENERAL POPULATION: WHAT IS THE PREDICTIVE ROLE OF RESILIENCE, COPING STYLE AND SOCIAL SUPPORT?

**DOI:** 10.1093/schbul/sby017.773

**Published:** 2018-04-01

**Authors:** David Mongan, Ciaran Shannon, Donncha Hanna, Adrian Boyd, Ciaran Mulholland

**Affiliations:** 1 Belfast Health and Social Care Trust; 2 Northern Health and Social Care Trust/ Queens University Belfast; 3 Queens University Belfast; 4 Northern Health and Social Care Trust

## Abstract

**Background:**

A history of childhood adversity is known to be associated with psychotic disorder as well as subclinical psychotic-like experiences. This study aimed to examine the relationship between specific types of childhood adversity and psychotic-like experiences in a general population sample, and to determine the predictive role of psychological resilience, coping style and perceived social support.

**Methods:**

An online survey was conducted with a US-based general population sample of 748 participants (aged 18 – 35 years) using Amazon’s Mechanical Turk (an online crowd-sourcing service). Participants completed the following validated measures: the Adverse Childhood Experiences Questionnaire (ACE-Q) as a measure of childhood adversities, the Prodromal Questionnaire (PQ-16) as a measure of psychotic experiences, the Brief Resilience Scale (BRS) measuring level of psychological resilience, the Brief COPE Scale as a measure of predominant coping style, the Multidimensional Scale of Perceived Social Support and the Neighbourhood Cohesion Scale. A series of backwards stepwise hierarchical regression analyses was employed to determine predictors of PQ-16 score.

**Results:**

Participants reported an average of 2.99 attenuated psychotic symptoms (from a total of 16 on the PQ-16), and an average of 2.77 childhood adversities (from a total of 10 on the ACE-Q). In the final regression model, which explained 33% of the variance in PQ-16 score, the specific types of childhood adversity which significantly predicted PQ-16 score were verbal abuse, sexual abuse and physical neglect. Level of resilience and coping via emotional support were significant negative predictive factors of PQ-16 score. The coping styles of self-distraction, denial, substance use, venting, religion and self-blame were significant positive predictors of PQ-16 score. Perceived social support and neighbourhood cohesion were not significant predictors.

**Discussion:**

The results of this study add support to the relationship between history of childhood adversity and psychotic-like experiences in the general population. Our data suggest that a differential effect exists dependent on the specific type of adversity (the strongest observed effect was for physical neglect). These findings highlight the need for routine clinical enquiry regarding childhood trauma for patients experiencing attenuated psychotic symptoms. We also found that psychological resilience and coping style were important predictive factors in this relationship (whilst perceived social support and neighbourhood cohesion were not). These may represent possible avenues for psychosocial augmentative interventions in the early stages of the psychosis continuum.

